# Clinical Diagnostic Assessment of Serum Testosterone in Intact and Castrated Tomcats †

**DOI:** 10.3390/vetsci13101081

**Published:** 2026-10-13

**Authors:** Koray Tekin, Maria Pereira, Laura Pimazzoni, Federico Bonsembiante, Paolo Zucchini, Stefano Romagnoli

**Affiliations:** 1 Department of Reproduction and Artificial Insemination, Faculty of Veterinary Medicine, Ankara University, 06110 Ankara, Türkiye; ktekin@ankara.edu.tr; 2 Department of Animal Medicine, Production and Health (MAPS), University of Padova, 35020 Legnaro, Italyfederico.bonsembiante@unipd.it (F.B.); paolo.zucchini@unipd.it (P.Z.)

**Keywords:** tomcat, testosterone, immunoassay, diagnostic accuracy, GnRH stimulation, anti-Müllerian hormone

## Abstract

Basal serum testosterone may help determine reproductive status in tomcats with an uncertain surgical history, but interpretation of a single result is limited by episodic secretion and reduced analytical precision at very low concentrations. We evaluated the analytical performance and diagnostic accuracy of an automated fluorescence enzyme immunoassay (FEIA) in 80 male cats (40 intact and 40 castrated). Within-run and between-day precision and dilutional recovery were assessed using pooled feline serum, and diagnostic performance was evaluated by receiver operating characteristic analysis. Testosterone concentrations were significantly higher in intact than castrated cats (*p* < 0.001), although overlap occurred at low concentrations. ROC analysis yielded an AUC of 0.773 (95% CI, 0.665–0.859). The Youden-derived threshold > 0.124 ng/mL had 60.0% sensitivity (95% CI, 43.3–75.1%) and 90.0% specificity (95% CI, 76.3–97.2%). No castrated cat had a concentration > 0.271 ng/mL; this cohort-specific rule-in threshold had 47.5% sensitivity (95% CI, 31.5–63.9%) and 100% observed specificity (95% CI, 91.2–100.0%). Therefore, clearly elevated basal testosterone can support the presence of functional testicular tissue, but concentrations ≤ 0.271 ng/mL cannot exclude intact status. Low or equivocal results require integration with clinical examination and, when indicated, GnRH stimulation and/or anti-Müllerian hormone measurement. Independent validation is required before broad clinical application.

## Simple Summary

Determining whether a male cat has functional testicular tissue can be challenging when its surgical history is unknown. This study evaluated serum testosterone concentrations in 40 intact and 40 castrated tomcats using an automated fluorescence enzyme immunoassay. Testosterone concentrations were higher overall in intact cats, although considerable overlap between groups occurred at low concentrations. No castrated cat in the present cohort had a testosterone concentration above 0.271 ng/mL, whereas 19 of 40 intact cats exceeded this value. Therefore, concentrations above 0.271 ng/mL may support the presence of functional testicular tissue, while lower concentrations cannot reliably distinguish intact from castrated cats. In cats with low or inconclusive basal testosterone concentrations, reproductive history and physical examination, including assessment of penile spines, should be considered together with additional hormonal testing, such as GnRH stimulation or anti-Müllerian hormone measurement when necessary. These findings demonstrate the potential clinical usefulness of basal testosterone measurement while emphasizing that the proposed thresholds require validation in independent feline populations.

## 1. Introduction

When the surgical history of a male cat is unavailable or uncertain, the clinically relevant question is whether functional testicular tissue is present. A central limitation of basal testosterone testing is the marked temporal variability of testosterone secretion in tomcats [1]. In a classic study of four mature intact tomcats sampled hourly over 24 h, testosterone showed episodic release without an evident diurnal rhythm and ranged from 0 to 23.5 ng/mL [2]. Consequently, a single blood sample collected during a secretory nadir may yield a very low or even undetectable concentration despite the presence of functional testes. This biological variability provides a direct explanation for why a single basal testosterone result may have limited sensitivity for identifying intact status.

Determining reproductive status is particularly important in cats with an uncertain neutering history, suspected cryptorchidism or retained testicular tissue, persistent male reproductive behaviour after reported castration, or disorders of sexual development [3,4,5,6]. Misclassifying an intact or cryptorchid cat as castrated may delay recognition of retained functional testicular tissue, whereas incorrectly assuming that a truly castrated cat is intact may lead to unnecessary additional investigation or exploratory surgery. Physical examination remains essential, but each component has limitations: ectopic or small retained testes may not be palpable; penile spines are androgen-dependent, and their absence may be difficult to interpret in young or hormonally suppressed cats; and imaging can be inconclusive when only a small amount of ectopic tissue is present [3,4,5,6,7,8]. These limitations create a practical need for laboratory tests that complement, rather than replace, the clinical examination.

Basal testosterone measurement is attractive because it is widely available, requires only one blood sample, and directly reflects Leydig-cell androgen production. Its main disadvantage is that low basal values cannot reliably exclude testicular tissue because of episodic secretion [2]. Dynamic testing provides an alternative: GnRH stimulation assesses the functional response of the pituitary–gonadal axis but requires administration of gonadorelin and repeat blood sampling; in adult tomcats, testosterone has been assessed before and 1 h after GnRH administration [9]. Anti-Müllerian hormone (AMH), a marker associated with Sertoli-cell-containing testicular tissue, may provide complementary information when basal testosterone and clinical findings remain inconclusive [10,11,12]. A clinically useful role for basal testosterone would therefore be as a simple first-line hormonal test if concentrations that provide strong rule-in evidence can be identified without implying that low values rule out intact status.

Accordingly, the primary objective of this study was to determine the diagnostic performance of a single basal serum testosterone measurement for distinguishing intact from castrated tomcats using the Tosoh AIA−360 FEIA and to identify assay-and cohort-specific decision thresholds for clinical interpretation. A secondary objective was to characterise within-run precision, between-day precision, and dilutional behaviour across high, intermediate, and very low testosterone concentrations. We hypothesised that a single basal testosterone measurement would provide high specificity but limited sensitivity for identifying functional testicular tissue, allowing a clinically useful rule-in threshold to be identified while leaving low concentrations non-definitive.

## 2. Materials and Methods

### 2.1. Study Population and Reference Standard

Serum samples from 80 male cats (40 intact and 40 castrated) were obtained retrospectively and prospectively at the Veterinary Teaching Hospital of the University of Padova. The clinical reference standard for reproductive status was established independently of the testosterone result using the criteria below. Samples originated from routine clinical or reproductive evaluation, and residual serum was used for the present study.

Intact tomcats (*n* = 40): Confirmed by bilateral scrotal testicular palpation, visual inspection of androgen-dependent penile spines, or direct surgical confirmation during elective orchidectomy.

Castrated tomcats (*n* = 40): Confirmed by verified medical records documenting orchidectomy or explicit owner verification combined with the complete absence of palpable testes and regression of penile spines.

In addition to the main clinical validation cohort, six stored serum samples from intact tomcats previously subjected to GnRH stimulation for reproductive purposes were included for analytical validation only. The archived aliquots used in the present study had been collected 1 h after intramuscular administration of 10 micrograms gonadorelin tetrahydrate (Cystoreline™, Ceva, Libourne, France, 50 micrograms/mL). Only these post-stimulation aliquots were used for the analytical-validation experiments; they were not included in the clinical validation cohort, ROC analysis, or reproductive-status threshold calculations. Cats aged ≥ 6 months were eligible for the clinical cohort. This criterion excluded younger kittens but included peripubertal males; because spermatogenic and endocrine maturation in male cats generally progresses through approximately 8–10 months of age [8], peripubertal status was considered explicitly in interpretation and as a study limitation. Blood samples were allowed to clot for 15 min and centrifuged at 2000× *g* for 10 min. Serum was separated and either analysed immediately or stored in aliquots at −20 degrees C until analysis. Samples demonstrating gross haemolysis, lipaemia, or icterus were excluded.

### 2.2. Hormone Assay and Instrumentation

Serum testosterone concentrations were measured using a competitive fluorescence enzyme immunoassay on an AIA−360™ automated analyser (Tosoh Bioscience, South San Francisco, CA, USA) using ST AIA-PACK^®^ Testosterone. In this assay, endogenous testosterone competes with the enzyme-labelled testosterone for antibody-binding sites, and the resulting fluorescence signal is inversely related to the testosterone concentration calculated from the manufacturer’s calibration curve. The minimum serum sample volume required was 300 µL [13].

Testosterone concentrations were initially obtained in ng/dL and converted to ng/mL for analysis (1 ng/dL = 0.01 ng/mL). Calibration was performed according to the manufacturer’s instructions using multi-point standard curves. Internal quality control samples were included in each analytical run to ensure assay performance. All reported testosterone concentrations, including analytical validation data, are presented in ng/mL following unit conversion.

### 2.3. Analytical Validation Protocols

Analytical performance was evaluated in accordance with established recommendations for immunoassay validation and previous veterinary evaluation of the FEIA platform and included assessment of within-run precision, between-day precision, and LUD [14,15].

Preparation of serum pools: Pooled serum was required because the precision experiments needed sufficient homogeneous material for repeated measurements at three concentration levels. For each pool, enough serum was prepared for 18 aliquots of 300 microL: 10 measurements for the within-run assessment and duplicate measurements on five working days for the between-day assessment, with the first two Day−1 measurements shared between the two schemes. The high-testosterone pool was prepared from serum of intact males, including GnRH-stimulated samples, selected for high concentrations. The intermediate pool was prepared from samples with approximately 0.5–2.0 ng/mL testosterone to obtain a target concentration around 1.0 ng/mL, and the low pool was prepared from low-testosterone serum from castrated cats. Available serum from the selected samples was combined to create each pool, mixed, divided into 300-microL aliquots, and stored at −20 degrees C until analysis.

Assay precision: To assess repeatability and between-day precision, three pooled serum samples representing approximately high (5.0 ng/mL), intermediate (0.9 ng/mL), and very low (0.04 ng/mL) testosterone concentrations were tested. The very-low-concentration pool was below the assay’s lower reportable limit of 0.07 ng/mL and was included only to characterise analyser behaviour at very low concentrations. Within-run precision was assessed by measuring each pool 10 times in the same analytical run. Between-day precision was assessed from duplicate aliquots on five consecutive working days. Variability was expressed as the coefficient of variation (CV). The first two Day−1 within-run measurements were also used as the Day−1 duplicates for the between-day assessment. The complete measurement scheme and raw values are provided in Supplementary Table S1.

Effect of sample dilution: To assess dilutional behaviour, serum from GnRH-stimulated intact tomcats was serially diluted with 0.9% saline to serum fractions of 100%, 90%, 80%, 70%, 60%, 50%, 40%, 30%, 20%, and 10%; a 0% serum tube containing saline alone served as the dilution blank. At each serum fraction, the measured testosterone concentration was compared with the concentration expected from proportional dilution. Recovery was calculated as measured concentration/expected concentration × 100. The exact dilution series and results are provided in Supplementary Table S3.

### 2.4. Statistical Analysis

Data were recorded in Microsoft Excel and analysed using (version 15.8; MedCalc Software Ltd., Ostend, Belgium). Descriptive statistics were calculated for all variables. Distributional assumptions were assessed with the Shapiro–Wilk test, and non-parametric methods were used where appropriate. Results are presented as mean + /− standard deviation or median and interquartile range according to data distribution. Age was available for all 80 cats. Body weight was available for 47 cats and was handled by complete-case analysis in the adjusted model; no imputation was performed. All 80 cats remained in the primary ROC analysis.

ROC curve analysis was used to evaluate the diagnostic performance of testosterone concentrations for differentiating intact from castrated cats [16]. The area under the curve (AUC) and 95% confidence intervals were calculated. The threshold providing the best statistical balance between sensitivity and specificity was determined using the Youden index. In addition, a clinically oriented rule-in threshold was evaluated based on the highest testosterone concentration observed in castrated cats. Values above this concentration were assessed as supporting the presence of functional testicular tissue. These thresholds were specific to the present cohort and were not intended to represent population reference intervals.

As an exploratory adjusted analysis, log10-transformed serum testosterone concentration was modelled as the dependent variable because the untransformed concentrations were right-skewed. Reproductive status was the primary explanatory variable, and age and body weight were included a priori as clinically plausible covariates that could influence testosterone concentration. The analysis used complete cases with body-weight data (*n* = 47; 21 intact and 26 castrated cats); missing body-weight values were not imputed. Model assumptions were evaluated by inspection of residual plots and homoscedasticity.

Cats with discordant classifications were reviewed descriptively. These included castrated cats with testosterone concentrations > 0.124 ng/mL and intact cats with concentrations ≤ 0.271 ng/mL. Available information on age, sampling date, confirmation of reproductive status, concurrent disease, medication history, time since castration, and repeat testosterone measurement was recorded. No additional inferential statistical analysis was performed for these subgroups. Individual case details are summarised in Supplementary Table S5.

## 3. Results

### 3.1. Study Population and Cohort Characteristics

The clinical diagnostic cohort comprised 80 male cats, including 40 intact and 40 castrated cats. Age was available for all cats, whereas body weight was available for 47 cats, including 21 intact and 26 castrated cats. Demographic and body-weight characteristics are summarised in Supplementary Table S2. Castrated cats were substantially older than intact cats (median [IQR], 6.37 [4.36–12.08] vs. 1.49 [0.83–2.97] years; *p* < 0.001). Body weight did not differ significantly between castrated and intact cats (4.50 [4.00–5.93] vs. 3.91 [3.50–5.50] kg, respectively; *p* = 0.294). Because of the marked age imbalance, age was retained as a covariate in the exploratory adjusted analysis and is considered further in the Discussion.

**Figure 1:**
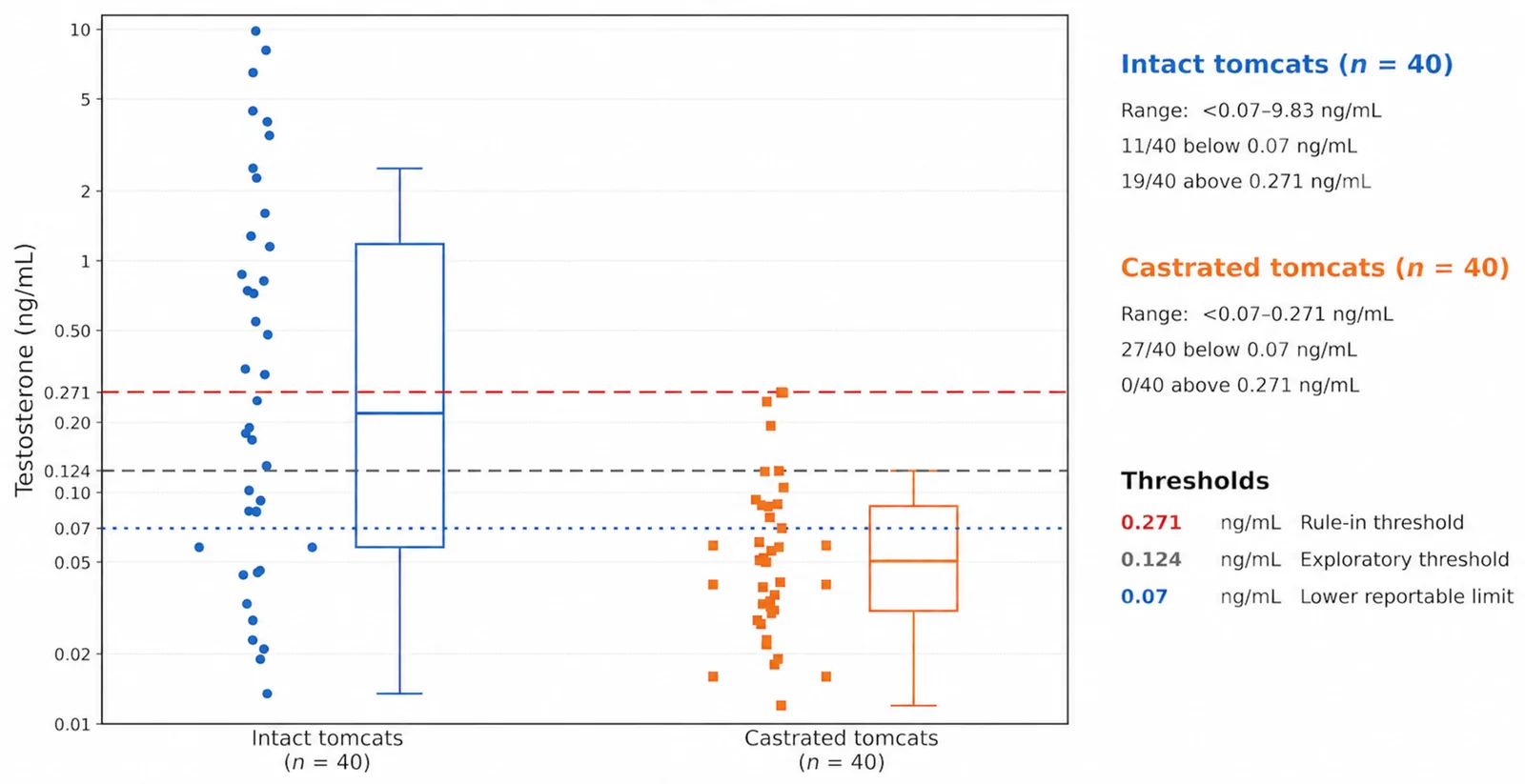
Distribution of serum testosterone concentrations in intact (*n* = 40) and castrated (*n* = 40) tomcats. The y-axis is displayed on a logarithmic scale. The horizontal line at 0.07 ng/mL indicates the assay’s lower reportable limit, the lower dashed line indicates the Youden-derived threshold of 0.124 ng/mL, and the upper dashed line indicates the high-specificity rule-in threshold of 0.271 ng/mL (100% observed specificity in this cohort). Each point represents one cat. Boxplots show the median, interquartile range, and 1.5 × interquartile range whiskers.

Serum testosterone concentrations were generally higher in intact compared to castrated cats, although partial overlap was observed at low concentrations (Figure 1). In intact cats, concentrations ranged from < 0.07 to 9.83 ng/mL; in castrated cats, concentrations ranged from < 0.07 to 0.271 ng/mL. The manufacturer’s instructions stated that instrument-generated values below 0.07 ng/mL were considered below the assay’s lower reportable limit and were not interpreted as exact quantitative concentrations. GnRH-stimulated samples showed markedly increased testosterone concentrations (5.55–14.29 ng/mL), confirming assay responsiveness across high physiological concentrations. Although some overlap was observed between GnRH-stimulated and non-stimulated intact cats, the highest testosterone concentrations occurred predominantly in GnRH-stimulated cats.

### 3.2. Analytical Validation

#### 3.2.1. Assay Precision

The assay gave the most consistent results at higher testosterone concentrations. At approximately 4.74 ng/mL, within-run and between-day CVs were 3.93% and 7.90%, respectively. At approximately 0.95 ng/mL, the corresponding CVs were 12.08% and 11.78%. Greater within-run variability was observed in the very-low pool, with a CV of 17.43%, whereas the between-day CV was numerically lower at 9.96%. Because the mean low-pool concentration (approximately 0.037–0.040 ng/mL) was below the 0.07 ng/mL reportable limit, neither CV should be interpreted as validated quantitative precision below that limit. In particular, the numerically lower between-day CV does not demonstrate superior between-day precision; it is best regarded as sampling variation in descriptive analyser-generated values below the validated reportable range (Table 1).

**Table 1:** Within-run and between-day precision of the FEIA testosterone assay in pooled feline serum.

Between-Day CV (%)	Between-Day Mean ± SD (ng/mL)	Within-Run CV (%)	Within-Run Mean ± SD (ng/mL)	Concentration Level
7.90	5.081 ± 0.401	3.93	4.743 ± 0.186	High pool
11.78	0.887 ± 0.105	12.08	0.950 ± 0.115	Intermediate pool
9.96	0.0396 ± 0.0039	17.43	0.0366 ± 0.0064	Low pool †

Abbreviations: CV, coefficient of variation; FEIA, fluorescence enzyme immunoassay; SD, standard deviation. † The mean concentration of the low pool was below the assay’s lower reportable limit of 0.07 ng/mL. These values are presented descriptively to indicate analyser variability at very low concentrations and should not be interpreted as validated quantitative precision below this limit.

#### 3.2.2. Effect of Sample Dilution

The difference between measured and expected concentrations increased with greater dilution. At the greatest dilution tested (1:10; 10% serum), the measured concentration was 2.308 ng/mL compared with an expected concentration of 1.259 ng/mL, corresponding to a recovery of approximately 183%. This indicates that highly diluted samples may overestimate testosterone concentration and should be interpreted cautiously (Supplementary Table S3).

### 3.3. Clinical Validation

#### ROC Curve Analysis

**Figure 2:**
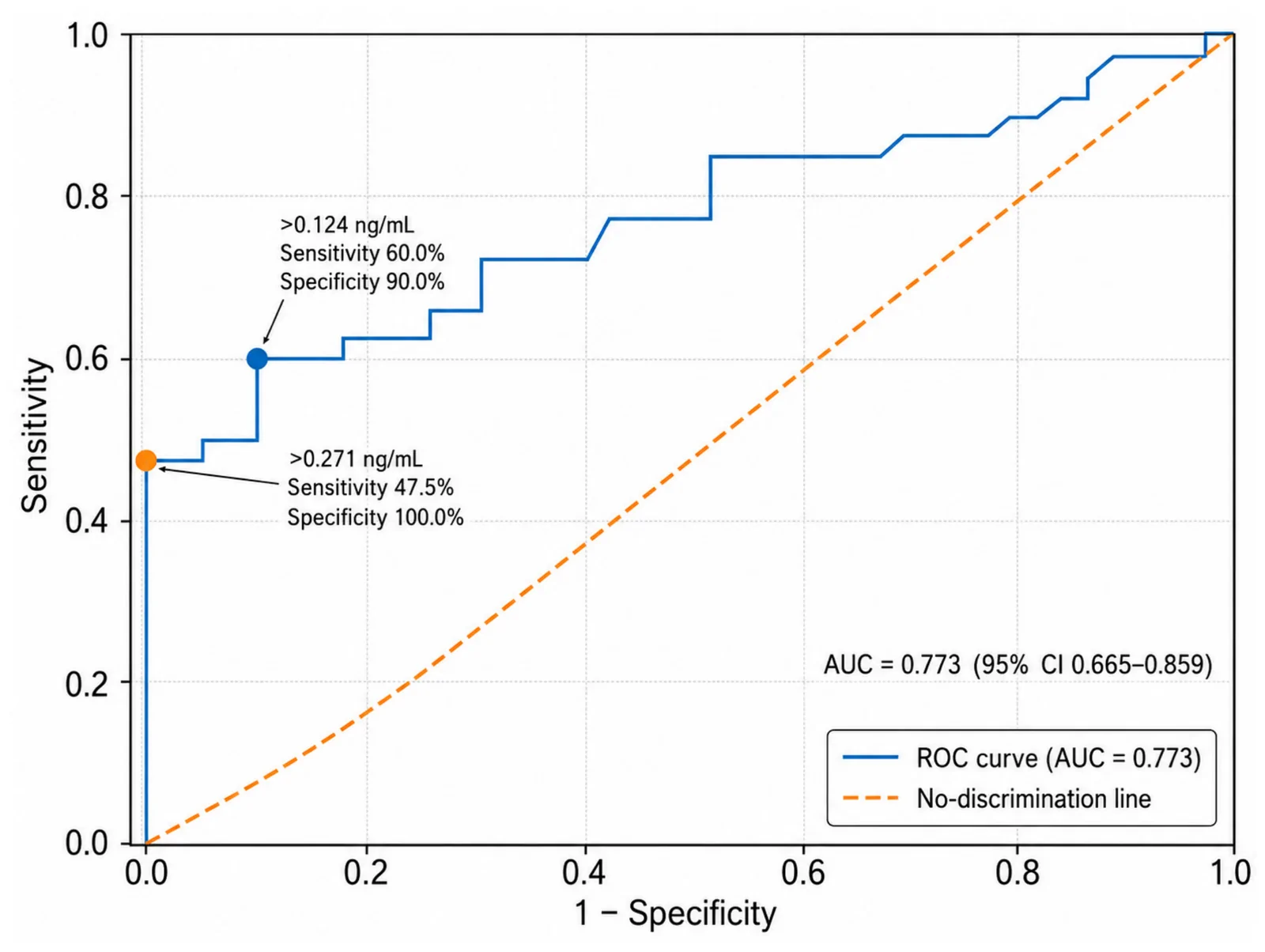
Receiver operating characteristic (ROC) curve for basal serum testosterone concentration in distinguishing intact and castrated tomcats. The ROC analysis showed that basal testosterone had moderate ability to distinguish intact from castrated tomcats. A concentration > 0.124 ng/mL provided the best overall balance between identifying intact cats and correctly excluding castrated cats. However, a value > 0.271 ng/mL was more useful for clinical confirmation: no castrated cat in this study had a testosterone concentration above this threshold. Because many intact cats had testosterone concentrations ≤ 0.271 ng/mL, a result below this threshold should not be interpreted as evidence that the cat is castrated.

ROC analysis demonstrated moderate discriminatory ability of basal serum testosterone concentration for differentiating intact and castrated tomcats (AUC = 0.773; 95% CI, 0.665–0.859; *p* < 0.0001) (Figure 2).

The optimal threshold identified by the Youden index was > 0.124 ng/mL, yielding a sensitivity of 60.0% and specificity of 90.0%. At this threshold, 24 of 40 intact cats were correctly classified as positive, whereas 4 of 40 castrated cats had concentrations above the threshold.

No castrated cat had a testosterone concentration > 0.271 ng/mL. This value was therefore evaluated as a cohort-specific high-specificity rule-in threshold. At this threshold, 19 of 40 intact cats were classified as positive, and all 40 castrated cats were classified as negative, corresponding to a sensitivity of 47.5% (95% CI, 31.5–63.9%) and an observed specificity of 100.0% (95% CI, 91.2–100.0%) (Table 2). Thus, more than half of the intact cats (21/40; 52.5%) did not exceed the rule-in threshold, and a result ≤ 0.271 ng/mL cannot be used to exclude functional testicular tissue.

The four castrated cats with concentrations > 0.124 ng/mL and the 21 intact cats with concentrations ≤ 0.271 ng/mL were reviewed individually (Supplementary Table S5). The four castrated cats had concentrations of 0.194–0.271 ng/mL. Low concentrations in intact cats occurred across a broad age range and in different seasons and clinical presentations. Two intact cats with initially low concentrations had higher testosterone concentrations in subsequently collected biological samples (0.323 and 1.280 ng/mL, respectively), illustrating that reproductive-status interpretation may change with the timing of a single basal sample.

Clinically, the two thresholds answer different questions. The > 0.124 ng/mL Youden-derived threshold provided the best statistical balance between sensitivity and specificity, whereas > 0.271 ng/mL provided stronger rule-in evidence because no castrated cat in this cohort exceeded it. Concentrations > 0.124 to ≤ 0.271 ng/mL should therefore be considered inconclusive. Concentrations ≤ 0.124 ng/mL favour castrated status statistically in this cohort but are not confirmatory, because intact cats also had values in this range. The thresholds are specific to this assay and cohort and are not population reference intervals.

**Table 2:** Diagnostic performance of basal serum testosterone thresholds for differentiating intact and castrated tomcats.

Specificity, % (95% CI)	Sensitivity, % (95% CI)	FP	TN	FN	TP	Threshold
90.0 (76.3–97.2)	60.0 (43.3–75.1)	4	36	16	24	Youden-derived threshold, > 0.124 ng/mL
100.0 (91.2–100.0)	47.5 (31.5–63.9)	0	40	21	19	High-specificity rule-in threshold, > 0.271 ng/mL

Abbreviations: CI, confidence interval; FN, false negative; FP, false positive; TN, true negative; TP, true positive. The > 0.271-ng/mL threshold showed 100% observed specificity in the present cohort.

In the exploratory complete-case multivariable model (*n* = 47), reproductive status remained independently associated with log10-transformed serum testosterone concentration after adjustment for age and body weight (intact vs. castrated: B = 0.823, SE = 0.235, 95% CI, 0.350 to 1.297; *p* = 0.001). Neither age (B = − 0.004, SE = 0.022, 95% CI, − 0.049 to 0.041; *p* = 0.867) nor body weight (B = 0.022, SE = 0.050, 95% CI, − 0.080 to 0.124; *p* = 0.668) showed an independent association. The model R2 was 0.312, and the adjusted R2 was 0.264. Full model results are provided in Supplementary Table S4.

## 4. Discussion

The main clinical finding of this study was that basal serum testosterone was more useful for confirming than excluding the presence of functional testicular tissue. No castrated cat in the present cohort had a concentration > 0.271 ng/mL, giving 100% observed specificity (95% CI, 91.2–100.0%). However, the same threshold had only 47.5% sensitivity (95% CI, 31.5–63.9%), meaning that 21 of 40 intact cats did not exceed it. The appropriate clinical interpretation is therefore asymmetric: a clearly elevated value can provide rule-in evidence of functional testicular tissue, whereas a low or borderline value cannot provide rule-out evidence.

This limited sensitivity has direct clinical consequences. If a result ≤ 0.271 ng/mL were interpreted as proof of castration, more than half of the intact cats in this cohort would have been incorrectly classified by that rule. In a cat with suspected cryptorchidism or uncertain surgical history, such an interpretation could delay recognition of retained functional testicular tissue; conversely, unresolved uncertainty may prompt unnecessary imaging or exploratory surgery in a truly castrated cat. Basal testosterone should therefore be used as one component of a stepwise diagnostic assessment rather than as a stand-alone exclusion test.

The limited sensitivity of basal testosterone is biologically plausible because testosterone secretion in tomcats is episodic and a single serum sample may be collected during a secretory nadir. Johnstone et al. documented marked within-day variation and episodic release in mature intact tomcats [2]. In the present study, 21 of 40 intact cats (52.5%) had testosterone concentrations ≤ 0.271 ng/mL, including 11 with analyser-generated values below the 0.07 ng/mL reportable limit. These findings reinforce that a single low basal testosterone result cannot reliably exclude functional testicular tissue.

The occurrence of higher testosterone concentrations in subsequent samples from two intact cats further supports temporal variation as the most plausible overall explanation. One cat had an initial concentration below 0.07 ng/mL followed by a concentration of 0.323 ng/mL in a separate sample collected the following day. Another cat had an initial concentration of 0.082 ng/mL and a subsequent concentration of 1.280 ng/mL. Although these were separate biological samples rather than analytical replicates, both subsequent results exceeded the high-specificity rule-in threshold of 0.271 ng/mL. These observations are consistent with episodic testosterone secretion and indicate that the reproductive-status interpretation of an intact cat may change depending on the timing of a single basal sample [2].

Age and incomplete sexual maturity may have contributed to low testosterone concentrations in some cats, but they did not explain the overall pattern. Among the 21 intact cats with concentrations ≤ 0.271 ng/mL, eight were young or peripubertal cats aged < 12 months, 12 were mature adults aged 1–8 years, and one was > 8 years. Male feline reproductive maturation develops progressively, with plasma testosterone increasing markedly at approximately 8 months and reproductive maturity generally reached between 8 and 10 months [8]. Thus, inclusion from 6 months of age may have increased the number of low results among peripubertal animals. Nevertheless, most low-testosterone cats were mature adults, and age was not independently associated with testosterone in the exploratory complete-case model (Supplementary Table S2). The substantial age difference between the intact and castrated groups remains a design limitation; a prospectively age-matched design would have reduced potential confounding more effectively than statistical adjustment alone.

Concurrent disease or an acute clinical presentation may also have influenced testosterone concentrations in some individuals. Fifteen of the 21 intact cats with concentrations ≤ 0.271 ng/mL had a concurrent clinical condition or acute presentation, including urinary, respiratory, neurological, musculoskeletal, gastrointestinal, and neoplastic disorders or trauma. Nevertheless, no single disease category predominated, disease severity was not standardised, and equivalent clinical information was not compared between intact cats with low and high testosterone concentrations. Consequently, the present data cannot establish whether concurrent illness contributed directly to the low results. This factor should therefore be regarded as a possible modifier rather than a demonstrated principal cause.

Exogenous hormonal suppression should also be considered in individual discordant cases. One intact cat with a testosterone concentration below 0.07 ng/mL had been included in a deslorelin-related study, although its treatment status at the time of sampling was unclear. Deslorelin can suppress pituitary–gonadal function and markedly reduce testosterone production and androgen-dependent characteristics in male cats [17,18]. If this cat had received deslorelin before blood collection, the result would have represented treatment-associated suppression rather than spontaneous variation in an untreated intact cat. This uncertainty warrants a sensitivity analysis rather than assuming that the sample is equivalent to the other intact cats.

Analytical performance at very low concentrations additionally limits interpretation of the lowest values. Eleven of the 21 intact cats with concentrations ≤ 0.271 ng/mL had results below 0.07 ng/mL, which was the lower boundary of the manufacturer’s reportable range. These instrument-generated values should therefore not be interpreted as exact quantitative concentrations. The greater variability observed in the low-concentration serum pool also indicates reduced consistency near the lower end of the assay response. This is consistent with recognised limitations of immunoassays at concentrations approaching or falling below their validated measuring range, where analytical variability and assay-dependent bias may become more influential [14].

Overall, the available evidence suggests that episodic testosterone secretion and the timing of a single basal sample were the principal contributors to the low values observed in intact cats. Peripubertal status, concurrent illness, and possible deslorelin exposure may have contributed in individual animals, while analytical variability at very low concentrations limited interpretation of the exact magnitude of results below 0.07 ng/mL. Advanced age and body weight did not explain the overall pattern. Low values occurred across different sampling seasons, but the study was not designed or sufficiently powered to evaluate seasonal effects formally. Previous work has examined variation in feline testosterone and reproductive characteristics under natural photoperiod, indicating that season remains a potential biological factor requiring standardised prospective evaluation [19,20].

**Figure 3:**
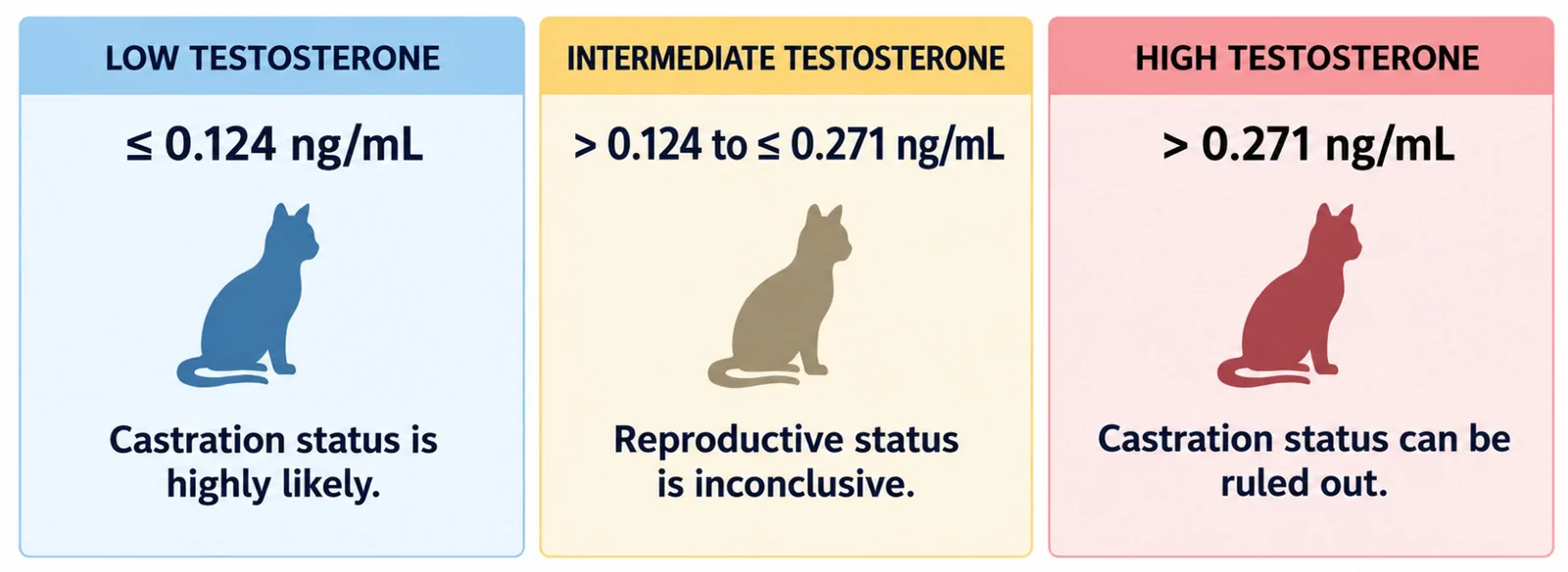
Clinical interpretation of basal serum testosterone concentrations in tomcats. A concentration ≤ 0.124 ng/mL is highly indicative of castration, although castration cannot be confirmed because low basal testosterone concentrations also occurred in intact cats. Concentrations > 0.124 to ≤ 0.271 ng/mL are considered inconclusive because basal testosterone alone cannot reliably determine reproductive status within this range. A concentration > 0.271 ng/mL strongly supports the presence of functional testicular tissue and rules out castration in the present cohort, as no castrated cat had a value above this threshold. For results ≤ 0.271 ng/mL, a stepwise approach is recommended: reproductive history and physical examination, particularly assessment of penile spines, should be considered first, followed by interpretation of basal testosterone and, when reproductive status remains uncertain, GnRH stimulation testing and complementary AMH measurement when required. This approach prioritises simple, readily available, and low-cost clinical assessment before more specialised hormonal testing.

In cats with uncertain reproductive status, a stepwise approach can minimise unnecessary testing while respecting the limitations of each method (Figure 3). Reproductive history should be assessed first, including reported castration, age at castration when known, previous hormonal treatment, and the cat’s age and sexual maturity. This should be followed by external genital examination, testicular palpation, and assessment of penile spines [1,5,7,8]. Basal serum testosterone can then be used as the first hormonal test; the AIA−360 assay requires a minimum serum volume of 300 microL [13]. A concentration > 0.271 ng/mL supports functional testicular tissue in this cohort, whereas concentrations ≤ 0.271 ng/mL remain non-definitive. When clinical examination and basal testosterone remain inconclusive, GnRH stimulation can provide a dynamic functional assessment [5,9], and AMH may provide complementary evidence of Sertoli-cell-containing testicular tissue when uncertainty persists, or stimulation testing is impractical [10,11,12].

The assay showed concentration-dependent imprecision, with greater variability as testosterone concentrations decreased. Because the low pool was below the 0.07 ng/mL reportable limit, its within-run CV of 17.43% and between-day CV of 9.96% are descriptive only. The lower numerical between-day CV should not be interpreted as a paradoxical improvement in precision; below the validated reportable range, small absolute differences, limited replicate numbers, and analyser rounding can produce unstable percentage CV estimates. In clinical practice, results below 0.07 ng/mL should therefore be reported as < 0.07 ng/mL rather than interpreted as exact quantitative values or used alone to confirm castration.

Dilutional recovery was close to the expected values at higher serum fractions of 70–90% but progressively deviated as the serum fraction decreased. At the highest dilution tested (1:10; 10% serum), the observed concentration was approximately 183% of the expected value, indicating that proportional recovery was not maintained across the full dilution series. Because saline was used as the diluent, matrix effects may have contributed to the increasing deviation. At the greatest dilution tested, recovery increased to approximately 183%, indicating loss of dilutional linearity and possible overestimation as testosterone concentrations decreased. However, because dilution also alters the serum matrix, this finding does not definitively demonstrate overestimation in naturally occurring low-concentration clinical samples. It should therefore be regarded primarily as an analytical characteristic of dilutional behaviour rather than as a limitation affecting interpretation of undiluted clinical samples or the proposed rule-in application.

This study has several limitations. The cohort was hospital-based and included both retrospectively stored and freshly collected samples during routine reproductive evaluation, which may limit generalisability to other clinical populations. Intact and castrated cats differed substantially in age; however, age was not independently associated with testosterone concentration in the exploratory adjusted analysis. Body weight was unavailable for some cats, thereby reducing the sample size of the secondary multivariable analysis, although this did not affect the primary ROC analysis. In addition, blood collection was not standardised according to the episodic pattern of testosterone secretion [2,21], and repeated basal samples were not routinely available. Sampling was also not standardised according to season or photoperiod; therefore, potential seasonal effects could not be formally evaluated [19,20]. Some younger cats may have been incompletely sexually mature, which could have contributed to low basal testosterone concentrations [8,22]. Specific clinical subgroups, including cats with confirmed cryptorchidism or retained testicular tissue, were not represented and should be evaluated separately in future studies. Finally, the > 0.271 ng/mL threshold was derived from the present cohort and showed 100% observed specificity (95% CI, 91.2–100.0%) but limited sensitivity (47.5%); therefore, independent validation in larger and clinically diverse populations is required before broad clinical application.

## 5. Conclusions

In conclusion, a single basal serum testosterone measurement can provide useful rule-in evidence when reproductive status is uncertain, but it cannot reliably rule out functional testicular tissue. In this cohort, concentrations > 0.271 ng/mL were observed only in intact cats, yielding 100% observed specificity (95% CI, 91.2–100.0%), but sensitivity was only 47.5% (95% CI, 31.5–63.9%). Therefore, concentrations ≤ 0.271 ng/mL should not be interpreted as confirmation of castration and should be integrated with reproductive history, physical examination, and, when necessary, GnRH stimulation and/or AMH measurement. The proposed thresholds are assay-and cohort-specific and require independent external validation.

## Data Availability

The original contributions presented in this study are included in the article/Supplementary Materials. Further inquiries can be directed to the corresponding authors.

## Supplementary Materials

The following supporting information can be downloaded at https://www.mdpi.com/article/10.3390/vetsci13101081/s1, Table S1. Raw measurement scheme and values used for within-run and between-day precision assessments; Table S2. Characteristics of intact and castrated tomcats included in the clinical validation cohort; Table S3. Dilution scheme and dilutional recovery of the GnRH-stimulated feline serum pool; Table S4. Multivariable linear regression of factors associated with log10-transformed serum testosterone concentration in tomcats; Table S5. Case-level characteristics of castrated tomcats above the Youden-derived threshold and intact tomcats at or below the rule-in threshold; Table S6. Case-level data for all 80 primary clinical observations included in the ROC analysis.
