# Supplementary material for: Clinical Diagnostic Assessment of Serum Testosterone in Intact and Castrated Tomcats
Source: Vet Sci. 2026 Oct 13;13(10):1081. doi: 10.3390/vetsci13101081 (PMC13709688; doi:10.3390/vetsci13101081)
Supplement: Supplementary file 1 [file vetsci-13-01081-s001.zip › vetsci-4587633-supplementary.pdf]

Supplementary Materials

Clinical Diagnostic Assessment of Serum Testosterone in Intact and Castrated Tomcats

Koray Tekin, Maria Pereira, Laura Pimazzoni, Federico Bonsembiante, Paolo Zucchini and Stefano Romagnoli

Supplementary Table S1. Raw measurement scheme and values used for within-run and between-day precision assessments.

Between-day mean ± SD, CV	Within-run mean ± SD (ng/mL)	Day 5 duplicate (ng/mL)	Day 4 duplicate (ng/mL)	Day 3 duplicate (ng/mL)	Day 2 duplicate (ng/mL)	Day 1 between-day duplicate (ng/mL)	Day 1 within-run measurements, n = 10 (ng/mL)	Pool
5.081 ± 0.401, 7.90%	4.743 ± 0.186, 3.93%	5.368, 6.038	4.876, 4.841	4.967, 5.057	5.252, 5.120	4.716, 4.570	4.923, 4.483, 4.713, 4.885, 4.762, 5.099, 4.562, 4.719, 4.716, 4.570	High
0.887 ± 0.105, 11.78%	0.950 ± 0.115, 12.08%	0.706, 0.857	0.852, 0.903	0.814, 1.047	0.724, 0.884	1.220, 0.862	0.888, 0.938, 0.841, 1.010, 1.007, 0.987, 0.905, 0.840, 1.220, 0.862	Intermedi-ate
0.0396 ± 0.0039, 9.96%	0.0366 ± 0.0064, 17.43%	0.040, 0.049	0.041, 0.044	0.041, 0.038	0.033, 0.039	0.030, 0.041	0.033, 0.030, 0.031, 0.036, 0.037, 0.039, 0.038, 0.051, 0.030, 0.041	Low

Values were converted from analyser-reported ng/dL to ng/mL by dividing by 100. Low-pool values were below the assay's lower reportable limit of 0.07 ng/mL and are shown only as descriptive analyzer-generated outputs; they should not be interpreted as validated quantitative precision below this limit.

Supplementary Table S2. Characteristics of intact and castrated tomcats included in the clinical validation cohort

P value	Castrated cats	Intact cats	Variable
—	40	40	Number of cats
<0.001	6.37 (4.36–12.08)	1.49 (0.83–2.97)	Age, years, median (IQR)
—	26	21	Body weight available, n
0.294	4.50 (4.00–5.93)	3.91 (3.50–5.50)	Body weight, kg, median (IQR)
—	0.90–9.00	1.40–10.40	Body weight, kg, range

Abbreviation: IQR, interquartile range. Age was available for all 80 cats. Body weight was available for 47 cats, including 21 intact and 26 castrated cats.

Supplementary Table S3. Dilution scheme and dilutional recovery of the GnRH-stimulated feline serum pool

Recovery (%)	Observed testosterone (ng/mL)	Expected testosterone (ng/mL)	Serum fraction (%)
100.0	12.5845	12.5845	100
101.2	11.4640	11.3261	90
105.8	10.6500	10.0676	80
106.0	9.3345	8.8092	70
114.5	8.6445	7.5507	60
122.0	7.6775	6.2923	50
127.8	6.4350	5.0338	40
143.9	5.4330	3.7754	30
160.5	4.0390	2.5169	20
183.4	2.3080	1.2585	10
Not applicable	NA	0	0

Expected testosterone concentrations were calculated from the concentration of the undiluted serum pool and the corresponding serum fraction. Recovery was calculated as observed concentration/expected concentration × 100. Dilutions were prepared using 0.9% saline. †The 0% serum tube contained only 0.9% saline and served as the dilution blank.

Supplementary Table S4. Multivariable linear regression of factors associated with log10-transformed serum testosterone concentration in tomcats

P value	95% CI	Standard error	Regression coefficient, B	Predictor
<0.001	−2.002 to −0.712	0.320	−1.357	Intercept
0.001	0.350 to 1.297	0.235	0.823	Reproductive status: intact versus castrated
0.867	−0.049 to 0.041	0.022	−0.004	Age, years
0.668	−0.080 to 0.124	0.050	0.022	Body weight, kg

Supplementary Table S5. Case-level characteristics of castrated tomcats above the Youden-derived threshold and intact tomcats at or below the rule-in threshold.

A. Castrated tomcats with basal testosterone >0.124 ng/mL

Repeat	Time since castration	Medication	Concurrent condition	Status confirmation	Date; season	Age (y)	Testosterone (ng/mL)	Cat ID
None reported	NR	NR	NR	Castrated status in hospital database; individual confirmation method NR	4 Apr 2023; spring	8.68	0.194	Cat 042
None reported	NR	NR	NR	Castrated status in hospital database; individual confirmation method NR	5 Apr 2023; spring	5.10	0.247	Cat 044
None reported	NR	NR	NR	Castrated status in hospital database; individual confirmation method NR	21 Jun 2023; summer	14.48	0.271	Cat 056
None reported	NR	NR	NR	Castrated status in hospital database; individual confirmation method NR	29 Jan 2024; winter	6.05	0.269	Cat 076

B. Intact tomcats with basal testosterone ≤0.271 ng/mL

Repeat measurement	Relevant medication/exposure	Condition/presentation	Reference-standard information	Date; season	Age (y)	Testosterone (ng/mL)	Cat ID
None reported	NR	Gastrointestinal system	Archived record‡	25 Jan 2022; winter	15.57	<0.07†	Cat 001
None reported	NR	Respiratory system	Archived record‡	11 Feb 2022; winter	0.84	0.102	Cat 002
None reported	NR	Deslorelin study	Archived record‡	18 Mar 2022; spring	3.01	<0.07†	Cat 005
None reported	NR	Trauma	Archived record‡	26 May 2022; spring	2.40	<0.07†	Cat 010
None reported	NR	Depressed sensorium	Archived record‡	30 May 2022; spring	2.25	0.130	Cat 011
None reported	NR	Musculoskeletal system	Archived record‡	7 Jun 2022; summer	0.93	0.180	Cat 012
Separate sample: 0.323 ng/mL on 3 Oct 2022	NR	Urinary system	Archived record‡	2 Oct 2022; autumn	1.49	<0.07†	Cat 014
None reported	NR	Neoplasia	Archived record‡	7 Jan 2023; winter	3.02	<0.07†	Cat 016
Separate sample: 1.280 ng/mL on 4 Apr 2023	NR	Urinary system	Archived record‡	11 Jan 2023; winter	0.78	0.082	Cat 017
None reported	NR	Neurological symptoms	Archived record‡	26 Mar 2023; spring	0.98	<0.07†	Cat 018
None reported	NR	Routine check-up	Archived record‡	26 Mar 2023; spring	5.23	<0.07†	Cat 019
None reported	NR	Neoplasia	Archived record‡	18 May 2023; spring	2.96	0.083	Cat 020
None reported	NR	Respiratory system	Archived record‡	6 Aug 2023; summer	0.50	0.169	Cat 024
None reported	NR	Neurological symptoms	Archived record‡	1 Sep 2023; autumn	0.58	<0.07†	Cat 025
None reported	NR	Routine check-up	Archived record‡	16 Oct 2023; autumn	0.59	<0.07†	Cat 026
None reported	NR	Urinary system	Archived record‡	18 Oct 2023; autumn	1.30	<0.07†	Cat 027
None reported	NR	Urinary system	Archived record‡	24 Nov 2023; autumn	1.73	0.190	Cat 028

Repeat measurement	Relevant medication/exposure	Condition/presentation	Reference-standard information	Date; season	Age (y)	Testosterone (ng/mL)	Cat ID
None reported	NR	Trauma	Archived record‡	11 Mar 2024; spring	2.19	0.249	Cat 031
None reported	NR	Routine check-up	Reproductive examination§	9 Sep 2024; autumn	5.15	0.092	Cat 035
None reported	NR	Routine check-up	Reproductive examination§	3 Oct 2024; autumn	1.25	0.0831	Cat 036
None reported	NR	Preoperative check-up before castration	Reproductive examination§	16 Oct 2024; autumn	0.54	<0.07†	Cat 037

NR, not recorded; † analyser-generated value below the 0.07 ng/mL lower reportable limit and therefore displayed as <0.07 ng/mL; ‡ archived clinical record; § Reproductive examination and blood samples collected freshly and analysed immediately. These data are provided for descriptive review of discordant cases and are not an additional inferential analysis.

Supplementary Table S6. Case-level data for all 80 primary clinical observations included in the ROC analysis.

Clinical presentation	Testosterone (ng/mL)	Date of blood collection	Breed	Age (y)	Reproductive status	Cat ID
Gastrointestinal system	<0.07†	25/01/22	European Shorthair	15.57	Intact	Cat 001
Respiratory system	0.102	11/02/22	European Shorthair	0.84	Intact	Cat 002
Respiratory system	9.83	16/02/22	European Shorthair	0.63	Intact	Cat 003
Fiv/Felv positive	0.723	03/03/22	European Shorthair	10.18	Intact	Cat 004
Deslorelin study	<0.07†	18/03/22	Bengala	3.01	Intact	Cat 005
Urinary system	3.48	22/03/22	European Shorthair	1.87	Intact	Cat 006
Urinary system	3.991	12/04/22	European Shorthair	0.86	Intact	Cat 007
Routine check-up	8.138	13/04/22	Norwegian Forest	4.28	Intact	Cat 008
FIP	0.547	18/05/22	European Shorthair	0.88	Intact	Cat 009
Trauma	<0.07†	26/05/22	European Shorthair	2.40	Intact	Cat 010
Depressed sensorium	0.13	30/05/22	European Shorthair	2.25	Intact	Cat 011
Musculoskeletal system	0.18	07/06/22	European Shorthair	0.93	Intact	Cat 012
Dental disease	1.603	07/09/22	Maine Coon	4.07	Intact	Cat 013
Urinary system	<0.07†	02/10/22	European Shorthair	1.49	Intact	Cat 014

Clinical presentation	Testosterone (ng/mL)	Date of blood collection	Breed	Age (y)	Reproductive status	Cat ID
Urinary system	0.323	03/10/22	European Shorthair	1.49	Intact	Cat 014
Musculoskeletal system	0.874	06/11/22	European Shorthair	0.52	Intact	Cat 015
Neoplasia	<0.07†	07/01/23	European Shorthair	3.02	Intact	Cat 016
Urinary system	0.082	11/01/23	European Shorthair	0.78	Intact	Cat 017
Neurological symptoms	<0.07†	26/03/23	European Shorthair	0.98	Intact	Cat 018
Routine check-up	<0.07†	26/03/23	European Shorthair	5.23	Intact	Cat 019
Preoperative check-up	1.28	04/04/23	European Shorthair	1.01	Intact	Cat 017
Neoplasia	0.083	18/05/23	European Shorthair	2.96	Intact	Cat 020
Megestrol acetate study	6.486	19/05/23	British Shorthair	1.03	Intact	Cat 021
Trauma	0.341	29/05/23	Maine Coon	0.79	Intact	Cat 022
Trauma	2.509	01/08/23	European Shorthair	2.58	Intact	Cat 023
Respiratory system	0.169	06/08/23	Ragdoll	0.50	Intact	Cat 024
Neurological symptoms	<0.07†	01/09/23	European Shorthair	0.58	Intact	Cat 025
Routine check-up	<0.07†	16/10/23	European Shorthair	0.59	Intact	Cat 026
Urinary system	<0.07†	18/10/23	European Shorthair	1.30	Intact	Cat 027
Urinary system	0.19	24/11/23	European Shorthair	1.73	Intact	Cat 028
FeLV positive	0.744	20/02/24	European Shorthair	1.64	Intact	Cat 029
Trauma	0.818	08/03/24	European Shorthair	3.18	Intact	Cat 030
Trauma	0.249	11/03/24	European Shorthair	2.19	Intact	Cat 031
Preoperative check-up prior to castration	1.151	20/03/24	European Shorthair	0.80	Intact	Cat 032
Preoperative check-up prior to castration	4.445	29/05/24	European Shorthair	0.61	Intact	Cat 033
Trauma	2.28	04/06/24	European Shorthair	4.43	Intact	Cat 034
Routine check-up	0.092	09/09/24	British Shorthair	5.15	Intact	Cat 035
Routine check-up	0.0831	03/10/24	British Shorthair	1.25	Intact	Cat 036

**Clinical presentation**	**Testosterone (ng/mL)**	**Date of blood collection**	**Breed**	**Age (y)**	**Reproductive status**	**Cat ID**
Preoperative check-up prior to castration	<0.07†	16/10/24	European Shorthair	0.54	Intact	Cat 037
Routine check-up	0.4796	17/10/24	British Shorthair	2.78	Intact	Cat 038
NR	0.089	29/08/22	European Shorthair	4.38	Castrated	Cat 039
NR	<0.07†	07/03/23	European Shorthair	6.18	Castrated	Cat 040
NR	<0.07†	09/03/23	European Shorthair	16.65	Castrated	Cat 041
NR	0.194	04/04/23	European Shorthair	8.68	Castrated	Cat 042
NR	<0.07†	05/04/23	Maine Coon	6.16	Castrated	Cat 043
NR	0.247	05/04/23	European Shorthair	5.10	Castrated	Cat 044
NR	0.088	12/04/23	European Shorthair	13.28	Castrated	Cat 045
NR	<0.07†	13/04/23	European Shorthair	2.70	Castrated	Cat 046
NR	<0.07†	20/04/23	Carthusian	4.30	Castrated	Cat 047
NR	<0.07†	21/04/23	European Shorthair	6.56	Castrated	Cat 048
NR	<0.07†	25/04/23	European Shorthair	1.90	Castrated	Cat 049
NR	<0.07†	27/04/23	European Shorthair	2.01	Castrated	Cat 050
NR	0.123	08/05/23	European Shorthair	17.74	Castrated	Cat 051
NR	<0.07†	08/05/23	European Shorthair	13.27	Castrated	Cat 052
NR	<0.07†	09/05/23	European Shorthair	3.35	Castrated	Cat 053
NR	<0.07†	09/05/23	European Shorthair	4.76	Castrated	Cat 054
NR	<0.07†	15/06/23	European Shorthair	5.87	Castrated	Cat 055
NR	0.093	19/06/23	European Shorthair	1.22	Castrated	Cat 017
NR	0.271	21/06/23	European Shorthair	14.48	Castrated	Cat 056
NR	<0.07†	23/06/23	European Shorthair	10.48	Castrated	Cat 057
NR	<0.07†	23/06/23	European Shorthair	13.48	Castrated	Cat 058
NR	0.078	26/06/23	European Shorthair	5.48	Castrated	Cat 059
NR	<0.07†	12/09/23	European Shorthair	16.71	Castrated	Cat 060
NR	<0.07†	18/09/23	European Shorthair	14.00	Castrated	Cat 061

Clinical presentation	Testosterone (ng/mL)	Date of blood collection	Breed	Age (y)	Reproductive status	Cat ID
NR	<0.07†	19/09/23	European Shorthair	10.72	Castrated	Cat 062
NR	<0.07†	22/09/23	European Shorthair	16.73	Castrated	Cat 063
NR	<0.07†	22/09/23	European Shorthair	5.48	Castrated	Cat 064
NR	<0.07†	26/09/23	European Shorthair	9.26	Castrated	Cat 065
NR	<0.07†	26/09/23	European Shorthair	14.50	Castrated	Cat 066
NR	<0.07†	14/10/23	European Shorthair	6.79	Castrated	Cat 067
NR	0.087	06/11/23	European Shorthair	1.35	Castrated	Cat 068
NR	0.07	10/11/23	European Shorthair	3.58	Castrated	Cat 069
NR	<0.07†	13/11/23	European Shorthair	0.67	Castrated	Cat 026
NR	<0.07†	15/11/23	European Shorthair	2.87	Castrated	Cat 070
NR	<0.07†	16/11/23	European Shorthair	5.30	Castrated	Cat 071
NR	<0.07†	22/11/23	European Shorthair	11.68	Castrated	Cat 072
NR	0.124	17/01/24	European Shorthair	10.05	Castrated	Cat 073
NR	0.105	25/01/24	European Shorthair	7.07	Castrated	Cat 074
NR	<0.07†	26/01/24	European Shorthair	8.91	Castrated	Cat 075
NR	0.269	29/01/24	Scottish Straight	6.05	Castrated	Cat 076

† Analyser-generated values below the 0.07 ng/mL lower reportable limit are displayed as <0.07 ng/mL. NR, not recorded/not available in the source table. The 80 observations comprise 40 intact and 40 castrated cats used in the primary ROC analysis.
